# An imaging dataset of cervical cells using scanning near-field optical microscopy coupled to an infrared free electron laser

**DOI:** 10.1038/sdata.2017.84

**Published:** 2017-07-11

**Authors:** Diane E. Halliwell, Camilo L.M. Morais, Kássio M.G. Lima, Júlio Trevisan, Michele R.F. Siggel-King, Tim Craig, James Ingham, David S. Martin, Kelly Heys, Maria Kyrgiou, Anita Mitra, Evangelos Paraskevaidis, Georgios Theophilou, Pierre L. Martin-Hirsch, Antonio Cricenti, Marco Luce, Peter Weightman, Francis L. Martin

**Affiliations:** 1 Centre for Biophotonics, LEC, Lancaster University, Bailrigg, Lancaster LA1 4YW, UK; 2 School of Pharmacy and Biomedical Sciences, Uclan, Fylde Rd, Preston PR1 2HE, UK; 3 Biological Chemistry and Chemometrics, Institute of Chemistry, Federal University of Rio Grande do Norte, Natal 59072-970 RN, Brazil; 4 Institute of Astronomy, Geophysics and Atmospheric Sciences, University of São Paulo, Cidade Universitária, R. do Matão, 1226 - Butantã, São Paulo - SP, 05508-090, Brazil; 5 University Department of Physics, The Oliver Lodge Laboratory, Cambridge St, Liverpool L69 7ZE, UK; 6 Accelerator Science and Technology Centre (ASTEC), STFC, Daresbury Laboratory, Keckwick Ln, Daresbury, Warrington WA4 4AD, UK; 7 Institute of Reproductive and Developmental Biology, Department of Surgery & Cancer, Faculty of Medicine, Imperial College, South Kensington Campus, London SW7 2AZ, UK; 8 West London Gynaecological Cancer Centre, Imperial College NHS Healthcare, Department of Obstetrics and Gynaecology, St Mary’s Hospital, Praed Street, London W2 1NY, UK; 9 Department of Obstetrics and Gynaecology, University of Ioannina, Panepistemioypole, Panepistemio, Ioannina, 45110 Greece; 10 St James University Hospital, Beckett St, Leeds LS9 7TF, UK; 11 Lancashire Teaching Hospitals NHS Foundation Trust, Sharoe Green Lane North, Fulwood, Preston PR2 9HT, UK; 12 Istituto di Struttura della Materia, Via del Fosso del Cavaliere, 100 - 00133 Rome (RM), Italy

**Keywords:** Infrared spectroscopy, Predictive markers, Super-resolution microscopy

## Abstract

Using a scanning near-field optical microscope coupled to an infrared free electron laser (SNOM-IR-FEL) in low-resolution transmission mode, we collected chemical data from whole cervical cells obtained from 5 pre-menopausal, non-pregnant women of reproductive age, and cytologically classified as normal or with different grades of cervical cell dyskaryosis. Imaging data are complemented by demography. All samples were collected before any treatment. Spectra were also collected using attenuated total reflection, Fourier-transform (ATR-FTIR) spectroscopy, to investigate the differences between the two techniques. Results of this pilot study suggests SNOM-IR-FEL may be able to distinguish cervical abnormalities based upon changes in the chemical profiles for each grade of dyskaryosis at designated wavelengths associated with DNA, Amide I/II, and lipids. The novel data sets are the first collected using SNOM-IR-FEL in transmission mode at the ALICE facility (UK), and obtained using whole cells as opposed to tissue sections, thus providing an ‘intact’ chemical profile. These data sets are suited to complementing future work on image analysis, and/or applying the newly developed algorithm to other datasets collected using the SNOM-IR-FEL approach.

## Background & Summary

Conventional infrared (IR) spectroscopy techniques, such as attenuated total reflection Fourier-transform infrared (ATR-FTIR) spectroscopy, are limited in spatial resolution by the effect of diffraction, defined as the interference of waves when they hit an obstacle or slit. This effect restricts the spatial resolution of FTIR to about half the wavelength of light or ~3 μm to 30 μm ref. (1), with the resolution being a measure of how closely the lines of an image can be resolved (i.e., the number of independent pixels per value per unit length).

Scanning near field optical microscopy (SNOM) belongs to a family of nanoscopic techniques that have shown potential in providing detailed information on cell topography and cytoplasmic structures. The technique has been used to determine the localisation of molecules within the cell membranes of prostate cancer cells^2^; to define the cell surface and internal structures of healthy and anomalous sperm^3^; and has demonstrated potential for single molecule imaging^4^. SNOM has a clear advantage over conventional IR microscopy in terms of spatial resolution because it is able to overcome the diffraction limit by the use of an apertured fibre optic scanning tip. However, SNOM requires relatively high photon intensities such as those provided by an IR free electron laser (IR-FEL). SNOM-IR-FEL enables the simultaneous collection of topography and optical features at scales not normally achieved with conventional IR techniques, to produce high quality, chemically-rich images at designated wavelengths with a spatial resolution of ~0.2 μm (refs 5,6).

In 2009, an IR oscillator FEL was installed into ALICE (Accelerators and Lasers in Combined Experiments), a specific accelerator test facility using the superconducting Energy Recovery Linac (ERL) at Daresbury Laboratory (Warrington, UK)^7^. Set-up, functionality and diagnostics of the ALICE IR-FEL laser have been extensively documented together with the results of the first lasing of ALICE being achieved at a wavelength of 8 μm (7,8,9). Further developments have included the FEL beam being carried to a diagnostics room, the optimization of the IR-FEL at different wavelengths and the coupling to a SNOM.

In 2013, SNOM-IR-FEL was used to investigate the chemical differences between non-dysplastic Barrett’s oesophagus and Barrett’s associated oesophageal adenocarcinoma^5^. Of note, is that researchers where blinded to the samples and were asked to identify them post experiment. The SNOM-IR-FEL images showed a large contrast in DNA and proteins/glycoproteins between the samples. Since elevated levels of DNA and proteins are associated with the development of cancer^10^, the experimenters were able to correctly identify cancer tissue from benign tissue^5^.

The optical chemical imaging data presented here were acquired in the context of a pilot study conducted in 2015, to determine the feasibility and utility of using SNOM-IR-FEL in transmission mode in the detection of the biophysical properties of cervical cell abnormalities. During our experiment, the IR-FEL at ALICE was tuneable over the range of 5.5 to 8.8 μm (~1,818 cm^−1^ to ~1,136 cm^−1^), which includes a number of biologically important biomarkers^11^ at designated wavenumbers or wavelengths within the ‘fingerprint’ region (1,800–900 cm^−1^). Previous work has shown that ATR-FTIR spectroscopy is able to diagnose underlying cervical disease and segregate grades of cervical dyskaryosis more precisely than conventional cytology based upon changes within the ‘fingerprint’ region^12,13^. We selected four wavenumbers/wavelengths from this region to explore with SNOM-IR-FEL (Table 1). Spectra were also collected using traditional ATR-FTIR biospectroscopy to investigate the differences between methods. The results of this pilot study have been published and evidence the promise of the SNOM-IR-FEL technique^14^.

The datasets presented here are novel for two reasons. Firstly, they are the first dataset collected using SNOM-IR-FEL in transmission mode at the ALICE facility. Secondly, the data were collected by imaging whole cells as opposed to tissue sections, thus providing an ‘intact’ chemical profile. These datasets are suited to complementing future work on image analysis, and/or applying the newly developed algorithm to other datasets collected using the SNOM-IR-FEL approach.

## Methods

The study was approved by the National Research Ethics Service Committee London—Fulham (Approval number 13/LO/0126), and conducted according to the principles of the Declaration of Helsinki and all other applicable national or local laws and regulations. All patients gave written informed consent before any protocol-specific procedure was performed.

### Patient recruitment

Patients (5 pre-menopausal, non-pregnant females, aged 25 to 45 years of age) were selected from a larger cohort of patients taking part in a larger study who were scheduled, if necessary, to undergo local cervical treatment at Imperial College NHS Healthcare Trust. Patients selected for this study were based on their cytology and histology typing (worse grade) to match a diagnosis of ‘normal’, squamous lesions (low-grade dyskaryosis and high-grade dyskaryosis), pre-invasive mixed lesions involving both squamous and glandular cells (squamous defined as cervical intraepithelial neoplasia 2 [CIN2]; glandular defined as high-grade cervical glandular intraepithelial neoplasia [HGCGIN]), or developed glandular lesions (adenocarcinoma). All samples were collected prior to treatment. Patients were anonymised and assigned a unique identifier.

We collected patient characteristics that included ethnicity, parity, smoking habits, antibiotic use within the last 2 weeks, phase in their cycle and use of contraception. The type of contraception and the time of their cycle (follicular or luteal) were documented. Medical and gynaecological history was collected for each patient including time since last sexual intercourse. For each patient, we collected data on the cytology, HPV DNA test and typing and histology, if available. Ethnicity was self-reported as Caucasian, Asian or Black. Women who were HIV or hepatitis B/C positive, women with autoimmune disorders, and women that received pessaries within 14 days of sampling were excluded. Women with a previous history of cervical treatment were also excluded.

### Cervical cells collection

A sterile, disposable speculum was inserted, without lubricant, and a cervical sample of ThinPrep, liquid-based cytology (LBC) was taken from the cervix (ThinPrep, HOLOGIC Inc., Bedford, USA). This was analysed for cytological diagnosis and HPV DNA test and typing. HPV DNA test and 16/18 genotyping was carried out according to manufacturer’s guidelines using the Abbott RealTime High Risk (HR) HPV assay on Abbott M2000 platform; a clinically validated *in vitro* polymerase chain reaction (PCR) assay with identification of HPV-16, −18 and 12 other HR HPV subtypes (31, 33, 35, 39, 45, 51, 52, 56, 58, 59, 66, 68)^15^. From the remaining methanol-based fluid, 1 ml was stored at 4 °C at the Centre for Biophotonics, Lancaster University, England, until preparation for SNOM-IR-FEL and ATR-FTIR analysis.

### Cytology and histology typing

In the UK, the NHS Cervical Screening Programme (NHSCSP) adopted the revised British Society Clinical Cytology (BSCC) terminology into their new guidelines for the classification of cervical cytology in 2013 (ref. 16). Outside of the UK, the Bethesda system is widely used^17^. A summary of both classification systems is presented in Supplementary Tables 1 and 2 for squamous and glandular lesions, respectively. An overview of how cervical cytology and histology are determined is provided, as are images for cervical cytology and histology for normal, low-grade dyskaryosis, high-grade dyskaryosis and squamous and glandular cervical cancers^18,19,20,21,22^.

### Slide preparation

Each sample was agitated to disperse the cell pellet, and then a 500-μl aliquot was collected from the 1 ml sample into a clean micro tube. The 500-μl aliquots were centrifuged at 2,000 r.p.m. for 5 min and the ThinPrep supernatant was aspirated from above the pellet to remove its spectral signature (i.e., the methanol fixative). Each sample was re-suspended in 500 μl of distilled H_2_O, agitated and centrifuged again. The supernatant was removed again and the wash step was repeated once more. For ATR-FTIR analysis, the final pellet was immersed in 100 μl of distilled H_2_O, agitated and dispensed onto IR-reflective glass slides (Low-E; Kevley Technologies Inc., Chesterland, OH, USA) in a uniform spread of whole cells and allowed to bench dry for a minimum of 24 h. Samples were then stored in a desiccator for a minimum of 48 h to remove any residual water before spectral analysis.

For SNOM-IR-FEL analysis, the remaining 500-μl aliquot was washed as described above. If the final pellet was small, it was suspended in 500 μl of distilled H_2_O, and larger pellets in 1,000 μl of distilled H_2_O. Each suspension was then agitated to disperse the pellet, and 5–6 drops added to a cytofunnel held in a cytoclip that had been pre-loaded with a BaF_2_ slide; (Crystan Ltd, Dorset, UK). Samples were spun at 3,000 r.p.m. for 5 min in a Cytospin 4 Cytocentrifuge (Thermo Fisher Scientific Inc., MA, USA) to disperse the cells in a single layer onto the slide. Slides were then housed in slide cartridges and kept in a desiccator until required.

### SNOM and IR-FEL experimental set-up

The experiments were performed on the IR-FEL beamline at the ALICE energy recovery linear accelerator at Daresbury Laboratory^7,8,9^. The wavelength of light from the FEL was selected by changing the undulator gap and, at the present accelerator settings, could be varied continuously from about 5.5 to 8.8 μm (~1,818 cm^−1^ to ~1,136 cm^−1^), a range which covers a number of biologically important absorption bands^11^. The IR-FEL operates at a macro-pulse width of ~10 μs and a repetition rate of 10 Hz, which limits, and determines, the rate of data collection. The IR light from the FEL was transported to the experimental area via an evacuated beamline and exited the beamline through a KBr window. The intensity of the FEL radiation was attenuated using a set of polarisers and focussed onto the sample. A CaF_2_ beam-splitter enabled the FEL radiation to be split so that approximately 80% went to the SNOM and 20% was used as a reference signal. The reference signal was monitored with a single-element pyro detector (Gentec-EO UM9B-BL-D0, Banbury, Oxon UK).

The general principal of operation for the SNOM used in these experiments has been previously described^6^. In brief, the scanning tip is a specially prepared infrared-transmitting Chalcogenide glass fibre, where one end is usually etched to a sharp tip. Gold is then evaporated onto the tip so that it covers all but the very end, forming an aperture of 0.1–1 μm in diameter through which the light is coupled and collected. The sample is rastered under the tip, using an x-y Piezo-scanner, whilst keeping the tip-to-sample shear-force constant through an electro-mechanic feedback. A single IR-FEL macro-pulse is used for each pixel of the images. The standard mode of operation for IR-SNOM is reflection, where the light approaches the sample at a grazing incidence angle of approximately 15° and the reflected light is collected by the fibre, transmitted through the fibre and detected using a liquid nitrogen cooled mercury-cadmium-telluride (MCT) detector.

For the measurements reported here, the data were acquired in transmission mode, where the sample was illuminated through the slide, and the light that was transmitted through the sample was collected by the fibre. The fibre was cleaved, rather than etched, so the entire 6-μm diameter fibre core was used to collect the infrared light signal; this enabled a direct comparison with standard IR techniques such as ATR-FTIR spectroscopy. The SNOM was incorporated into an inverted optical microscope, which was used to locate specific cells of interest on the sample and to position them within the SNOM scan area.

A BaF_2_ slide containing the cells was mounted onto the SNOM and scans acquired at fixed wavelengths of 5.71 μm/~1,750 cm^−1^ (lipids), 6.06 μm/~1,650 cm^−1^ (Amide I), 6.46 μm/~1,550 cm^−1^ (Amide II) and 8.16 μm/~1,225 cm^−1^ (DNA-asymmetric phosphate stretching vibrations) for each set of cells. Each SNOM data set was collected at a one IR-FEL wavelength. The tip height (topography, or TOPO), raw transmission intensity (SNOM) and an IR-FEL intensity reference signal (ZERO) were simultaneously collected for each pixel, yielding three images to comprise one SNOM dataset. During data acquisition, two independent datasets were sequentially collected. For each row of the image, the ‘forward’ set was collected whilst the sample was moving in one direction and then the ‘backward’ set whilst the sample was moving in the opposite direction; then the same was repeated for each row of the image.

### ATR-FTIR spectroscopy

Spectra were acquired using a Tensor 27 FTIR spectrometer with a Helios ATR attachment (Bruker Optik GmbH). Each spectrum comprised 32 scans at 8 cm^−1^ wavenumber spacing with 2x interferogram zero-filling. Before the spectra were taken, the crystal was cleaned with distilled H_2_O and inspected by video camera to be free of any contaminants. A background spectrum was acquired before the sample slide was mounted and the stage moved to bring the cervical cells in contact with the diamond. Spectra were collected from ten random sites on the slide. Spectra were converted to absorbance by Bruker OPUS software (Bruker Inc., Billerica, MA, USA).

### Pre-processing of SNOM-IR-FEL image datasets

The raw forward and backward SNOM transmission image datasets were loaded into the freely available software Gwyddion 2.40, available at http://gwyddion.net/, and converted into text files ready for importing into MATLAB. No other pre-processing was performed other than file conversion; the images were not corrected for IR-FEL intensity variations. A second set of raw data files were converted into jpgs following image enhancement using Gwyddion; these files were used for the images of the data shown in this work.

### SNOM-IR-FEL image enhancement

**Figure 1:**
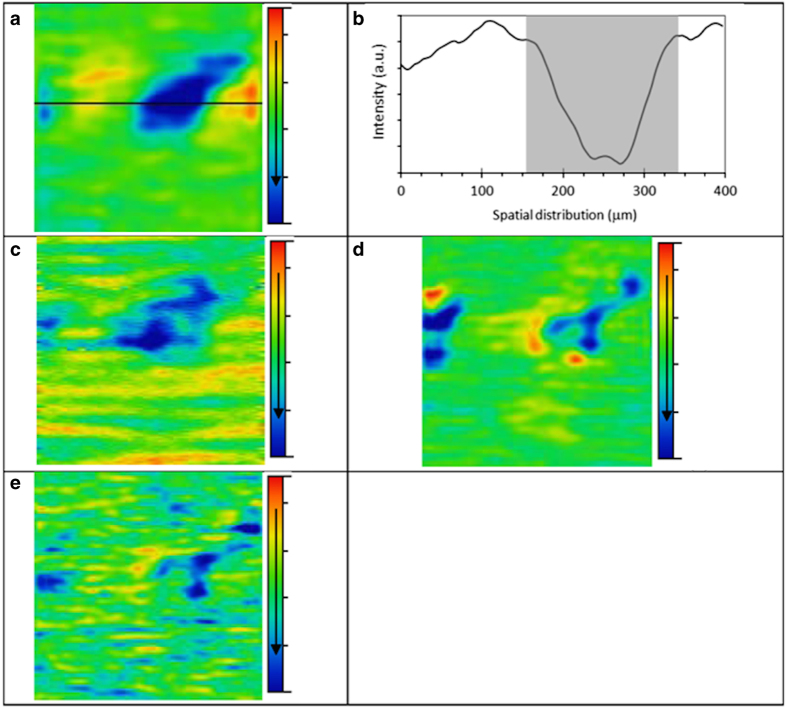
Transmission IR-SNOM images (400 μm×400 μm) of the same cell sampled from pre-invasive lesion (CIN2, HGCGIN) at the different biomarker wavelengths. (**a**) Amide I—6.06 μm (~1,650 cm^−1^), the horizontal line in (**a**) is shown in cross section in (**b**), (**c**) Amide II—6.46 μm (~1,550 cm^−1^), (**d**) DNA—8.16 μm (~1,225 cm^−1^) and (**e**) Lipids—5.71 μm (1,750 cm^−1^). The colour scale bar arrow indicates decreasing biomarker signal transmission, meaning increasing biomarker absorption. The shaded region in (**b**) corresponds to the interval selected for area calculation according to the cell content. CIN2, HGCGIN: Cervical intra-epithelial neoplasia, high-grade cervical glandular intraepithelial neoplasia.

The images presented in Fig. 1 were processed for presentation using Gwyddion 2.40. Median height line correction in the horizontal (fast scan) axis was applied, followed by the removal of high frequency noise using a two-dimensional Fourier-transform.

### Computational analysis.

#### SNOM-IR-FEL transmission image datasets

The raw SNOM transmission image datasets were processed using MATLAB software 2014a (Mathworks Inc., Natick, MA, USA) and PLS Toolbox version 7.9.3 (Eigenvector Research, Inc., WA, USA). For each cell studied, a set of four raw SNOM transmission image data files, corresponding to the four wavelengths used (e.g., each biomarker response), were imported into MATLAB as four matrixes with size of 150×150 (Fig. 1). To obtain a spectrum-like signal profile from the biomarker response, the biomarker data matrix was converted into a vector by the mean calculation of the matrix in the column-mode direction (equation (1)), where, s_j_ is an element of the row-vector s {1×150}, corresponding to the spectrum-like signal; *m* is the size of the image on column-mode direction; and x_ij_ is an element of the biomarker matrix X.

(1)sj=1m∑i=1mxij

Thereafter, the spectrum-like signal was normalized by mean-centring and absolute value. The bar charts were made with the area of the spectrum-like signal integrated into an interval of spatial distribution according to the cell content position (Fig. 1).

**Figure 2:**
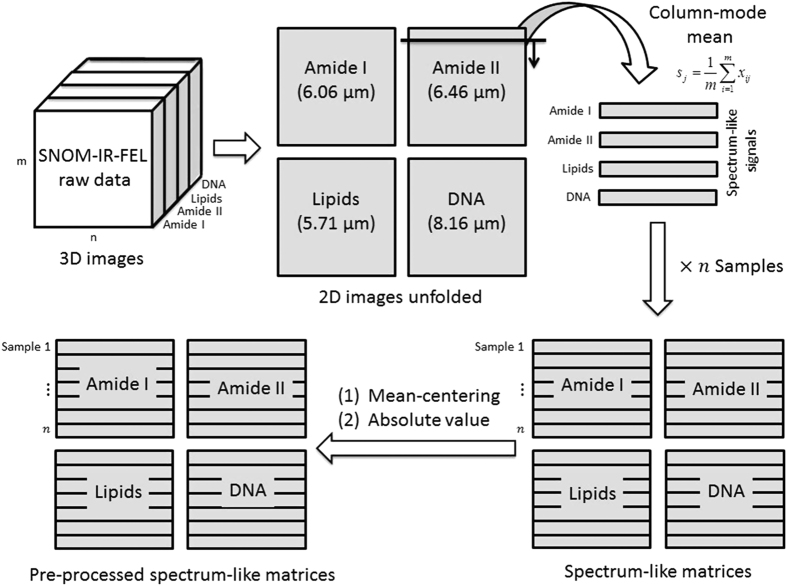
The computational steps taken in processing the data.

Principal component analysis (PCA) was performed with the whole spectrum-like signal using only mean-centring and absolute value as pre-processing. A summary of the computational steps in processing the data is given in Fig. 2. PCA is an unsupervised technique commonly used as the first step in analysing large, multivariate datasets. Unsupervised techniques require no information from the user but rely instead on an internal criterion to guide learning. In unsupervised learning, the system forms clusters (groupings, regions of data space). In general terms, PCA reduces the dimensionality of large datasets and using mathematical projection, the original dataset which may have involved many variables, can often be interpreted in just a few variables (i.e., the principal components; PCs). This reduced dimensional data set will allow the user to spot trends, patterns and outliers in the data, far more easily than would have been possible without performing the PCA. When applied to spectra, PCA identifies common sources of variance across spectra and collates them into a small number of dimensions. PCA is often not enough to segregate out data classes or clusters sufficiently. By applying a supervised technique such as Linear Discriminant Analysis (LDA) to the PCA output, it promotes inter-class variation to be identified whilst preventing over-fitting of the data.

PCA was executed using the average signal of each biomarker (triplicate) for five samples, one for each type of cell morphology: normal, low-grade dyskaryosis, high-grade dyskaryosis, CIN2, HGCGIN and adenocarcinoma Stage 1B1. Additionally, the area for each biomarker for each cell type was determined, as was the percentage area variation from ‘normal’ for each biomarker for each cell type.

#### ATR-FTIR spectra

The ATR-FTIR data were analysed using multivariate techniques of PCA for preliminary data reduction, and the output was processed using LDA and a variable selection technique employing Successive Projections Algorithm (SPA)^23^, in conjunction with LDA for selecting an appropriate subset of wavenumbers for classification purposes. SPA is a variable selection technique specifically designed to improve the conditioning of multiple linear regression by minimizing collinearity effects in the calibration data set and can result in models with good prediction ability^24^.

The classic Kennard-Stone (KS) uniform sampling algorithm^25^ was adopted to divide the available samples into training (70%), validation (15%) and prediction sets (15%) for construction and validation of the PCA-LDA and SPA-LDA models. The training set was used to obtain model parameters (including variable selection for LDA), and the validation set was employed to choose the best number of the PCs for PCA model and to guide the variable selection. The optimum number of variables for SPA-LDA was used to select variables employing the G function as cost function^25^.

#### Code availability

Morais, C.L.M. & Lima, K.M.G. Source code for: An imaging dataset of cervical cells using scanning near-field optical microscopy coupled to an infrared free electron laser. *Figshare:* https://dx.doi.org/10.6084/m9.figshare.3479003.v1 (2016).

## Data Records

The data is available through the following link under the collection name DataScienceSNOMSets.zip (Data Citation 1).

### Demographic data

These are considered as metadata, informing the imaging data. Fully documented patient characteristics are available for 4 out of the 5 patients; limited demography is available for the patient typed with high-grade dyskaryosis. There is one metadata.pdf summarizing the data and one metadata.doc file. Both of these files can be located in the folder named ‘metadata.’

### SNOM-IR-FEL imaging data. 

#### Raw data

The raw SNOM datasets are available as AFM files and are located in the folder labelled ‘Collated_FB_SNOM_Raw,’ with sub-folders containing data specific to individual patients and labelled as ‘Normal,’ ‘Low_Grade_Dyskaryosis,’ ‘High_Grade_Dyskaryosis,’ ‘Mixed_Lesion_CIN2_HGCGIN,’ and ‘Adenocarcinoma_Stage1B1.’ Each file is numbered to include the patient ID together with labelling for either the forward or backward image data. For example, the data file ‘0195-C21211-BSNOM.AFM’, has the prefix of 0195 which refers to the sequential numbering in which the images were collected. C212 refers to the patient ID followed by the area ‘II’ (one of one). The direction of the scan is indicated with the next letter: F for forward and B for backward. This is then followed by four letters indicating the type of data. SNOM is the raw SNOM transmission data, TOPO is the raw topography (e.g., 0195-C21211-FTOPO.AFM) and ZERO is the raw IR-FEL intensity reference signal data (e.g., 0195-C21211-FZERO.AFM). The data analysis described in this paper uses only the SNOM image data files; the TOPO and ZERO files are included for completion and would, in general, be used for other types of analysis.

The data contained in the SNOM and ZERO files are separately proportional to the intensity of the detected light within a given dataset. The absolute values are attenuated by the polarisers, the detectors, the box car integrators and by the Analogue-to-Digital converters; the polarisers and box car integrator sensitivity are adjusted separately for SNOM and ZERO for each dataset (a set of data with the same sequential number). Therefore, each signal has a different offset and scaling factor. Most of the data contained in the header files was input manually; the exceptions are the first 3 lines and the last 5 lines. The provision of header files is under development and these files were not meant for publication at this early stage. Whilst the operators endeavoured to make sure the data was correct, it is inevitable that sometimes parameters were not changed when they should have been. As the work reported in this paper did not use most of the information in the header files, no attempt was made to check to accuracy of the data therein.

#### Converted SNOM imaging data to txt files

Raw SNOM transmission data from each patient is also available as a txt file (converted from the raw AFM files) using Gwyddion 2.40. The txt file, can be opened in many applications including MATLAB, to reveal a matrix of values that are proportional to the intensity of the FEL light being detected for a given image; lower voltages correspond to less intensity of the FEL light being detected at that point in the sample. Each file is numbered as above, replacing the ‘AFM’ extension with a txt extension. These data are stored in a folder labelled ‘Collated_FB_SNOM_TXT,’ and individual patient data is collated into sub-folders labelled as described above (Table 1). To complement the above, some of the experimental parameters for each SNOM image are collated together into one convenient Excel spreadsheet called ‘SNOM_Data_Spreadsheet,’ which details where possible, scan steps, scan size and FEL power amongst others.

#### ATR-FTIR spectroscopy data

Data for each patient is available as an OPUS binary format file and collated into patient-specific sub-folders as described above (Table 2). Each file extension contains the date on which the data were collected. These data can be readily accessed and worked with via IRootlab^26^ (available at http://trevisanj.github.io/irootlab/).

## Technical Validation

### Samples

We included a sample collected from a patient with normal cervical cytology and infected with HPV ‘other type’ (i.e., not high-risk oncogenic types 16 or 18). As such, this provided a reference sample to compare the other samples with various grade of lesions, some of which were infected with high-risk oncogenic types.

### Cytology and histology typing

Images for cervical cytology and histology of normal, squamous and glandular lesions are provided^18,19,20,21,22^.

### Atomic force microscopy (AFM) imaging of cells

To evidence that whole cervical cells had been used to collect the SNOM-IR-FEL data, atomic force microscopy (AFM) was performed on the adenocarcinoma stage 1B1 sample, using a Bruker Innova AFM in contact mode using silicon nitride probes of nominal spring constant 0.07 Nm^−1^. Topography and deflection (error signal) channels were recorded simultaneously. The contact force of the AFM tip on the cells was minimised to optimise image quality. These AFM images have been previously reported^14^.

### SNOM-IR-FEL

No optical structures were observed in the reference signal. SNOM data sets were collected from the normal sample as a reference point in order to distinguish optical data distribution for the different types of cervical lesions.

### Computational analysis

The SNOM-IR-FEL reduced data were evaluated according to the following statistical parameters: variance, s.e., sample bias and mean squared error (MSE). The statistical results for the forward and backward mode are shown in Tables 3 and 4, respectively. The variance, s.e., bias and MSE were very small showing almost no variation between the various cells of the same type that were imaged. The data is almost unbiased to its mean, which shows the reproducibility of the reduced data between replicates of cells collected in different positions on the sample slide. The SNOM images used for analysis have been previously reported^14^.

These same statistical parameters for whole ATR-FTIR spectra are provided in Table 5. As can be seen, the variance, s.e., bias and MSE for ATR-FTIR data were also very small, showing almost no variation between replicates of spectra and unbiased trend.

## Additional Information

**How to cite this article:** Halliwell, D. E. *et al.* An imaging dataset of cervical cells using scanning near-field optical microscopy coupled to an infrared free electron laser. *Sci. Data* 4:170084 doi: 10.1038/sdata.2017.84 (2017).

**Publisher’s note:** Springer Nature remains neutral with regard to jurisdictional claims in published maps and institutional affiliations.

## Supplementary Material



Supplementary Tables

## Figures and Tables

**Table 1:** Patients′ information with cytology and histology typing, number of cells imaged at each wavenumber (cm^−1^)/wavelength (μm) by SNOM-IR-FEL and associated data folders containing either ‘raw’ forward and back SNOM images, topography and a FEL intensity reference signal and converted AFM files into txt files.

**Subjects (cytology & histology type) (subject ID)**	**Number of cells imaged by SNOM-IR-FEL**	**Wavenumber (cm**^**−1**^**) (wavelength, μm)**	**Tentative assignment of biomarker**	**Data set folders: ‘Collated_FB_SNOM-Raw’ & ‘Collated_FB_SNOM_TXT’**
Normal (MK212)	16	~1,225 (8.16)	DNA—asymmetric phosphate	Sub-folder: Normal
		~1,550 (6.46)	Amide II of proteins predominantly in *β* sheet conformation	
		~1,650 (6.06)	Amide I of proteins predominantly in α helix conformation	
		~1,750 (5.71)	Lipids	
Low-grade dyskaryosis (MK177)	6	As above	As above	Low_Grade_Dyskaryosis
High-grade dyskaryosis (MK126A)	2	As above	As above	High_Grade_Dyskaryosis
CIN2, HGCGIN (MK162)	5	As above	As above	Mixed_Lesion_CIN2_HGCGIN
Adenocarcinoma Stage 1B1 (MK238)	5	As above	As above	Adenocarcinoma_Stage1B1
Additional demographic data is available within the data set (metadata.pdf).				
CIN2, HGCGIN: Cervical intraepithelial neoplasia 2, high-grade cervical glandular intraepithelial neoplasia: SNOM-IR-FEL: Scanning near-field optical microscopy coupled with an electron free laser.				

**Table 2:** Patients′ information with cytology and histology typing, and the number and location of scans collected by ATR-FTIR spectroscopy.

**Subjects (cytology & histology type); (subject ID)**	**Number of scans collected by ATR-FTIR spectroscopy**	**Data set folder: ATR_FTIR**
Normal (MK212)	10	Sub-folder: Normal
Low-grade dyskaryosis (MK177)	10	Sub-folder: Low_Grade_Dyskaryosis
High-grade dyskaryosis (MK126A)	10	Sub-folder: High_Grade_Dyskaryosis
CIN2, HGCGIN (MK162)	10	Sub-folder: Mixed_Lesion_CIN2_HGCGIN
Adenocarcinoma Stage 1B1 (MK238)	10	Sub-folder: Adenocarcinoma_Stage1B1
ATR-FTIR: Attenuated total reflection, Fourier-transform spectroscopy; CIN2, HGCGIN: Cervical intraepithelial neoplasia 2, high-grade cervical glandular intraepithelial neoplasia.		

**Table 3:** Average variance, s.e., sample bias and mean squared error (MSE) for SNOM transmission image data in forward mode.

**Cell type (subject ID)**	**Biomarker**	**Variance**	**s.e.**	**Bias**	**MSE**
Normal (MK212)	Amide I*	7.77×10^−21^	3.60×10^−11^	−1.29×10^−26^	1.30×10^−21^
	Amide II†	2.31×10^−21^	1.60×10^−11^	−5.74×10^−27^	2.56×10^−22^
	DNA‡	1.15×10^−21^	1.20×10^−11^	−1.78×10^−26^	1.44×10^−22^
	Lipids§	6.83×10^−22^	1.07×10^−11^	1.94×10^−26^	1.14×10^−22^
Low-grade dyskaryosis (MK177)	Amide I*	4.44×10^−22^	1.05×10^−11^	0.00	1.10×10^−22^
	Amide II†	3.17×10^−22^	1.03×10^−11^	8.62×10^−27^	1.06×10^−22^
	DNA‡	1.74×10^−22^	6.60×10^−12^	0.00	4.36×10^−23^
	Lipids§	1.14×10^−21^	1.69×10^−11^	3.23×10^−27^	2.86×10^−22^
High-grade dyskaryosis (MK126)	Amide I*	2.14×10^−23^	3.27×10^−12^	1.29×10^−26^	1.07×10^−23^
	Amide II†	3.25×10^−23^	4.03×10^−12^	−1.29×10^−26^	1.62×10^−23^
	DNA‡	3.31×10^−21^	2.35×10^−11^	−6.46×10^−27^	5.52×10^−22^
	Lipids§	2.13×10^−21^	3.26×10^−11^	1.29×10^−26^	1.06×10^−21^
CIN2, HGCGIN (MK162)	Amide I*	7.05×10^−22^	1.88×10^−11^	1.29×10^−26^	3.53×10^−22^
	Amide II†	1.34×10^−24^	1.16×10^−12^	0.00	1.35×10^−24^
	DNA‡	3.45×10^−22^	9.29×10^−12^	−1.29×10^−26^	8.63×10^−23^
	Lipids§	9.99×10^−22^	2.24×10^−11^	−1.29×10^−26^	5.02×10^−22^
Adenocarcinoma Stage 1B1 (MK238)	Amide I*	1.28×10^−21^	1.79×10^−11^	−3.23×10^−27^	3.20×10^−22^
	Amide II†	1.94×10^−21^	2.20×10^−11^	9.69×10^−27^	4.84×10^−22^
	DNA‡	3.97×10^−20^	7.53×10^−11^	−4.06×10^−26^	5.67×10^−21^
	Lipids§	6.68×10^−21^	4.72×10^−11^	−4.31×10^−27^	2.23×10^−21^
All parameters are represented as relative absorbance.					
CIN2, HGCGIN: Cervical intraepithelial neoplasia 2, high-grade cervical glandular intraepithelial neoplasia.					

*Amide I: 1,650 cm^−1^.

^†^Amide II: 1,550 cm^−1^.

^‡^DNA: 1,225 cm^−1^.

^§^Lipids: 1,750 cm^−1^.

**Table 4:** Average variance, s.e., sample bias and mean squared error (MSE) for SNOM transmission image data in backward mode.

**Cell type (Subject ID)**	**Biomarker**	**Variance**	**s.e.**	**Bias**	**MSE**
Normal (MK212)	Amide I*	7.36×10^−21^	3.50×10^−11^	8.62×10^−27^	1.22×10^−21^
	Amide II†	1.75×10^−21^	1.58×10^−11^	1.29×10^−26^	2.50×10^−22^
	DNA‡	1.19×10^−21^	1.22×10^−11^	3.23×10^−27^	1.49×10^−22^
	Lipids§	9.95×10^−22^	1.29×10^−11^	4.31×10^−27^	1.66×10^−22^
Low-grade dyskaryosis (MK177)	Amide I*	6.86×10^−22^	1.51×10^−11^	0.00	2.28×10^−22^
	Amide II†	3.10×10^−22^	1.02×10^−11^	0.00	1.04×10^−22^
	DNA‡	2.36×10^−22^	7.68×10^−12^	−6.46×10^−27^	5.90×10^−23^
	Lipids§	1.48×10^−21^	2.22×10^−11^	8.62×10^−27^	4.93×10^−22^
High-grade dyskaryosis (MK126)	Amide I*	2.03×10^−23^	3.19×10^−12^	1.29×10^−26^	1.02×10^−23^
	Amide II†	3.04×10^−23^	3.90×10^−12^	1.30×10^−26^	1.52×10^−23^
	DNA‡	1.97×10^−21^	1.81×10^−11^	−2.37×10^−26^	3.28×10^−22^
	Lipids§	2.04×10^−21^	3.19×10^−11^	−6.46×10^−27^	1.02×10^−21^
CIN2, HGCGIN (MK162)	Amide I*	3.41×10^−21^	3.37×10^−11^	−4.31×10^−27^	1.14×10^−21^
	Amide II†	1.49×10^−24^	1.22×10^−12^	0.00	1.49×10^−24^
	DNA‡	2.08×10^−22^	8.32×10^−12^	−8.62×10^−27^	6.92×10^−23^
	Lipids§	9.92×10^−22^	2.23×10^−11^	6.46×10^−27^	4.97×10^−22^
Adenocarcinoma Stage 1B1 (MK238)	Amide I*	1.28×10^−21^	2.07×10^−11^	8.62×10^−27^	4.28×10^−22^
	Amide II†	1.91×10^−21^	2.19×10^−11^	−1.29×10^−26^	4.80×10^−22^
	DNA‡	3.99×10^−20^	7.54×10^−11^	1.48×10^−26^	5.69×10^−22^
	Lipids§	6.85×10^−21^	4.78×10^−11^	0.00	2.28×10^−21^
All parameters are represented as relative absorbance.					
CIN2, HGCGIN: Cervical intraepithelial neoplasia 2, high-grade cervical glandular intraepithelial neoplasia.					

*Amide I: 1,650 cm^−1^.

^†^Amide II: 1,550 cm^−1^.

^‡^DNA: 1,225 cm^−1^.

^§^Lipids: 1,750 cm^−1^.

**Table 5:** Variance, s.e., bias and average mean squared error (MSE) for replicates of ATR-FTIR spectra.

**Cell type (Subject ID)**	**Variance**	**s.e.**	**Bias**	**MSE**
Normal (MK212)	9.88×10^−11^—1.80×10^−6^	3.14×10^−6^—4.24×10^−4^	−1.42×10^−8^—1.11×10^−8^	9.88×10^−12^—1.80×10^−7^
Low-grade dyskaryosis (MK177)	1.95×10^−10^—7.62×10^−7^	4.42×10^−6^—2.76×10^−4^	−8.94×10^−9^—1.19×10^−8^	1.95×10^−11^—7.62×10^−8^
High-grade dyskaryosis (MK126)	5.55×10^−10^—1.67×10^−6^	7.45×10^−6^—4.08×10^−4^	−1.27×10^−8^—1.34×10^−8^	5.55×10^−11^—1.67×10^−7^
CIN2, HGCGIN (MK162)	2.92×10^−10^—7.02×10^−7^	5.40×10^−6^—2.65×10^−4^	−1.04×10^−8^—1.04×10^−8^	2.92×10^−11^—7.02×10^−8^
Adenocarcinoma Stage 1B1 (MK238)	2.53×10^−10^—7.35×10^−7^	5.03×10^−6^—2.71×10^−4^	−1.27×10^−8^—1.64×10^−8^	2.53×10^−11^—7.34×10^−8^
All parameters are represented as absorbance units.				
CIN2, HGCGIN: Cervical intraepithelial neoplasia 2, high-grade cervical glandular intraepithelial neoplasia.				
