# Supplementary material for: An imaging dataset of cervical cells using scanning near-field optical microscopy coupled to an infrared free electron laser
Source: Sci Data. 2017 Jul 11;4:170084. doi: 10.1038/sdata.2017.84 (PMC5505104; doi:10.1038/sdata.2017.84)
Supplement: Supplementary Tables [file sdata201784-s2.pdf]

Supplementary Information

An imaging dataset of cervical cells using scanning near-field optical microscopy coupled to an infrared free electron laser

Diane E. Halliwell[1,12], Camilo L. M. Morais[2], Kássio M. G. Lima[2], Julio Trevisan[3], Michele R. F. Siggel-King[4,5], Tim Craig[4], James Ingham[4], David S. Martin[4], Kelly Heys[1], Maria Kyrgiou[6,7], Anita Mitra[6,7], Evangelos Paraskevaidis[8], Georgios Theophilou[9], Pierre L. Martin-Hirsch[1,10], Antonio Cricenti[11], Marco Luce[11], Peter Weightman[4], & Francis L. Martin[1,12]

Cytology		Histology
**NHSCSP 2013[18] (UK cytology system)**	**Bethesda 2014[19] (cytology system widely used outside of the UK)**	**Used widely throughout the world**
Borderline changes in squamous/endocervical (glandular) cells	ASCUS<br>ASC-H	HPV
Low-grade dyskaryosis	LGSIL	CIN1
High-grade dyskaryosis (moderate)	HGSIL	CIN2
High-grade dyskaryosis (severe)	HGSIL	CIN3
High-grade dyskaryosis/?invasive SCC	HGSIL/SCC	SCC

Supplementary Table 1. Cytology and Histology classification systems in the UK and outside of UK for squamous lesions. ASCUS: Atypical squamous cells of undetermined significance; ASC-H: Atypical squamous cells that cannot exclude HGSIL; CIN: Cervical intraepithelial neoplasia; HGSIL: High-grade intraepithelial lesion; HPV: Human papillomavirus; LGSIL: Low-grade intraepithelial lesion; SCC: Squamous cell carcinoma. N.B.: Terms may include 'G' in the grade (e.g., LGSIL) or omit it (e.g., LSIL).

NHSCSP 2013[18]<br>(UK cytology system)	Bethesda 2014[19]<br>(cytology system widely used outside of the UK)
Borderline changes in endocervical cells	Atypical glandular cells, not otherwise specified<br>a. Endocervical<br>b. Endometrial<br>c. Glandular
?Glandular neoplasia<br>a. ?Glandular neoplasia of endocervical type (divided into low-grade cervical glandular intraepithelial neoplasia [LG-CGIN], and high-grade cervical glandular intraepithelial neoplasia [HG-CGIN])<br>b. ?Glandular neoplasia (non-cervical)	Atypical glandular cells favour neoplastic<br>a. Endocervical<br>b. Glandular
	Endocervical adenocarcinoma *in situ*
	Adenocarcinoma<br>a. Endocervical<br>b. Endometrial<br>c. Extra uterine<br>d. Not otherwise specified

Supplementary Table 2. Cytology classification systems in the UK and outside of UK for glandular lesions. LG-CGIN: Low-grade cervical glandular intraepithelial neoplasia; HG-CGIN: High-grade cervical glandular intraepithelial neoplasia. Histology classification for glandular lesions are described in detail by Zaino (2000).
